# Evolution of a hybrid zone of two willow species (*Salix* L.) in the European Alps analyzed by RAD‐seq and morphometrics

**DOI:** 10.1002/ece3.9700

**Published:** 2023-01-04

**Authors:** Pia Marinček, Loïc Pittet, Natascha D. Wagner, Elvira Hörandl

**Affiliations:** ^1^ Department of Systematics, Biodiversity, and Evolution of Plants (with Herbarium) University of Göttingen Göttingen Germany

**Keywords:** hybridization, introgression, morphology, next‐generation sequencing, population genomics

## Abstract

Natural hybridization of plants can result in many outcomes with several evolutionary consequences, such as hybrid speciation and introgression. Natural hybrid zones can arise in mountain systems as a result of fluctuating climate during the exchange of glacial and interglacial periods, where species retract and expand their territories, resulting in secondary contacts. Willows are a large genus of woody plants with an immense capability of interspecific crossing. In this study, the sympatric area of two diploid sister species, *S. foetida* and *S. waldsteiniana* in the eastern European Alps, was investigated to study the genomic structure of populations within and outside their contact zone and to analyze congruence of morphological phenotypes with genetic data. Eleven populations of the two species were sampled across the Alps and examined using phylogenetic network and population genetic structure analyses of RAD Seq data and morphometric analyses of leaves. The results showed that a homoploid hybrid zone between the two species was established within their sympatric area. Patterns of genetic admixture in homoploid hybrids indicated introgression with asymmetric backcrossing to not only one of the parental species but also one hybrid population forming a separate lineage. The lack of F1 hybrids indicated a long‐term persistence of the hybrid populations. Insignificant isolation by distance suggests that gene flow can act over large geographical scales. Morphometric characteristics of hybrids supported the molecular data and clearly separated populations of the parental species, but showed intermediacy in the hybrid zone populations with a bias toward *S. waldsteiniana*. The homoploid hybrid zone might have been established via secondary contact hybridization, and its establishment was fostered by the low genetic divergence of parental species and a lack of strong intrinsic crossing barriers. Incomplete ecological separation and the ability of long‐distance dispersal of willows could have contributed to the spatial expansion of the hybrid zone.

## Graphical abstract

We detected a hybrid zone of willows in the European Alps by using population genomics and morphometrics. Introgression and broad geographical scale characterize this hybrid zone.

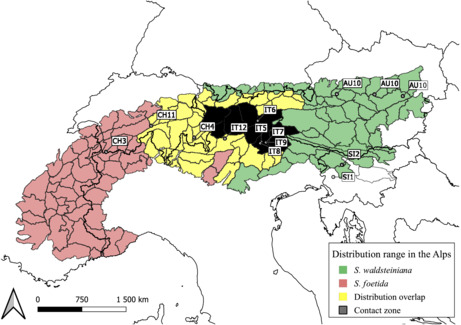

## 1 INTRODUCTION

Hybridization is described as an interbreeding of individuals from two or more populations, which are distinguishable on the basis of at least one heritable character (Arnold, 1997; Harrison, 1990). Hybridization was appraised as a widespread phenomenon among flowering plants (Stebbins, 1959). Several studies from the late 20th and early 21st centuries have revealed that angiosperms tend to hybridize frequently with an estimated 3%–25% of observed hybridization events spread unevenly across 40% of families and 16% of genera (Mallet, 2005; Whitney et al., 2010).

Areas where hybridization naturally occurs are described as hybrid zones (Harrison, 1993). Production of a viable first generation of hybrids (F1), despite pre‐ and postzygotic incompatibilities of divergent taxa, is usually the most difficult step (Arnold, 1997; Mallet, 2005). A rise in ploidy levels of hybrids – allopolyploidization – often results in chromosomal or genetic incompatibilities between hybrids and parents, while homoploid hybrids are less likely to follow this path (Mallet, 2007; Rieseberg, 1997; Soltis et al., 2014; Soltis & Soltis, 2009). Hybridization can boost genetic variance and, consequently, hybrids might be capable of inhabiting different ecological niches than their parental species (Mallet, 2007). Thus, ecological selection might become the driver for reproductive isolation of genetically compatible parental and admixed lineages. Homoploid offspring that are not reproductively isolated allow for backcrossing with one or both of their parents (Mallet, 2007; Yakimowski & Rieseberg, 2014). Such an outcome of hybridization produces a wide array of recombinant types, described as a hybrid swarm (Harrison, 1993). This reticulate exchange of interspecific genetic material through hybrid offspring is described with the term “introgression” (Anderson, 1949) and can result in genetic, ecological, phenotypical, or other changes in hybridizing populations (Arnold, 1997; Mallet, 2007). Introgression is a well‐documented process in flowering plants (Anderson, 1949, Stebbins, 1950, Stebbins, 1959, reviewed in Yakimowski & Rieseberg, 2014). High levels of introgressive hybridization in nature are often associated with disturbances that open new ecological niches (reviewed in Yakimowski & Rieseberg, 2014). Several studies on adaptive trait introgression of plants suggested that homoploid hybrid variants could directly be involved in important ecological adaptations (reviewed in Kadereit, 2015, Yakimowski & Rieseberg, 2014).

Studies of plant hybrids revealed that F1 hybrids showed a mosaic of intermediate, as well as parental morphological traits, which could be explained by the dominant inheritance patterns of certain characters (Rieseberg et al., 1993). Moreover, first, as well as later‐generation hybrids, frequently exhibit transgressive (extreme, compared to parental) morphological characteristics (Rieseberg et al., 1999). Patterns of the dominance of morphological traits were found to be hard to predict even in F1 hybrids, exhibiting a mismatch of characters with dominance in conflicting directions (Thompson et al., 2021). Conclusively, hybrid morphology can be diverse, exceeding the simple assumption of intermediacy, and should be studied case specific.

In a spatio‐temporal context, many hybrid zones are a result of a secondary contact of diverging populations that were separated during the last glacial maximum (LGM), between 30 and 18 thousand years ago (Ivy‐Ochs et al., 2008). In Europe, during this time, the unfavorable conditions forced the species to retreat to southern refugia on the Iberian, Apennine, and Balkan Peninsulas, as well as the Caucasus and Caspian Sea regions, from where they rapidly moved northward, once the conditions were favorable (Hewitt, 1999). Mountain regions, such as the European Alps, act as a range expansion barrier, resulting in a saturation of many hybrid zones between different populations (Hewitt, 2004). Most hybrid zones have remained relatively spatially stable since their establishment hundreds or thousands of years ago (Harrison, 1990). Until now most hybrid zone studies in the Alps were conducted on single contact zone instances and only a few studies focused on populational studies over larger ranges of species' contact zones. Such studies give a more conclusive picture of the extent of hybrid zones and general patterns of admixture and processes underlying them.

The genus *Salix* L., with about 450 species distributed worldwide, is the largest genus in the willow family (Salicaceae) (Argus, 1997). Willows exhibit many different growth forms, ranging from large trees to tiny dwarf shrubs, and inhabit a wide range of habitats, including the unfavorable mountain and arctic regions, where they are one of the dominating arborescent plant groups (Skvortsov, 1999). In Europe, *Salix* is the largest woody plant genus with about 65 described species (Rechinger, 1964), 33 of which were described in the mountain systems of the European Alps (Aeschimann & Lauber, 2004). However, for systematic biologists, willows have always posed a difficulty. One of the troubling taxonomic and phylogenetic aspects of this genus is frequent hybridization. High crossing ability across the genus and even among distantly related species has been examined with experimental crossings (Argus, 1974; Wichura, 1865) and observed in nature (e.g., Gramlich et al., 2018; Hardig et al., 2000; Neumann, 1981; Oberprieler et al., 2013; Wagner et al., 2021). Flexible pollination by insects, as well as facultatively by wind, and long‐distance dispersal of airborne seeds enable high gene flow between individuals. This, on the account of the absence of reproductive barriers, can result in hybridization (Hörandl et al., 2012). Favoring of permeable interspecific gene flow to enhance genetic variability in combination with long‐distance dispersal is linked to willows' ecological role to colonize open niches as pioneer species (Hörandl et al., 2012). Particularly closely related taxa of the same ploidy level are likely to naturally form homoploid hybrids (Mosseler, 1990). Homoploid hybridization of mountain willows is frequently linked to species coming into secondary contact after glacier retreat. Several studies that have examined hybrid zones of different *Salix* species pairs detected patterns of introgression (Fogelqvist et al., 2015; Gramlich et al., 2016, 2018; Gramlich & Hörandl, 2016; Hardig et al., 2000). Hybrid individuals were also observed to evolve adaptive growth forms and exhibited higher tolerances to certain abiotic conditions (Gramlich et al., 2016, 2018). However, even though this species‐rich genus is well known for many hybridizing taxa with frequently reported hybrid phenotypes in nature, they were rarely examined in detail and over a broader geographical scale (Hörandl, 1992). Many hybrid combinations in willows remain single, infertile F1 individuals without further evolution. Moreover, quite often the morphological variants of pure species were misidentified as hybrids (Neumann, 1981). Traditional molecular markers like DNA sequences of barcoding markers (internal transcribed spacer of the nuclear ribosomal DNA, or plastid markers like petD or matK) failed to resolve interspecific relationships (e.g., Azuma et al., 2000; Hardig et al., 2010; Lauron‐Moreau et al., 2015; Percy et al., 2014; Wu et al., 2015), and therefore studies on willow hybrid zones remained relatively scarce. Only in the last decade, RAD‐seq approaches (Baird et al., 2008) were proven to be a powerful tool to disentangle phylogenetic relationships of *Salix* clades and species (Wagner et al., 2018, 2020), radiation patterns (He et al., 2021), as well as genetic structure of natural hybrid populations (Gramlich et al., 2018). The huge number of SNPs generated by this method allows for detailed molecular analyses of closely related sister species and their hybrids.

**FIGURE 1:**
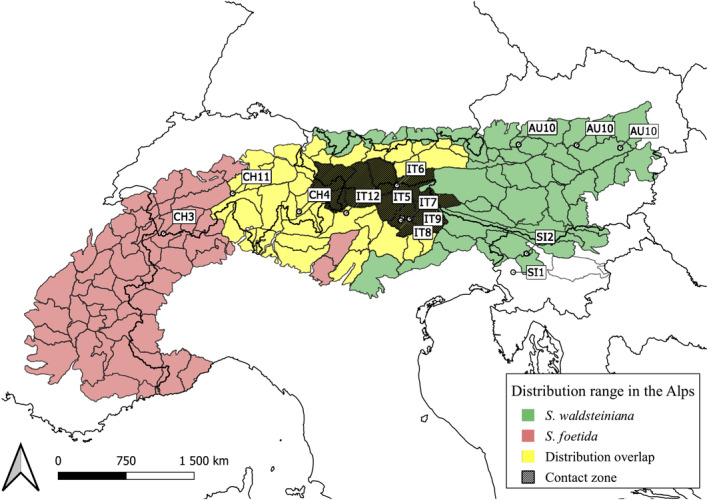
Distribution map of *S. foetida* and *S. waldsteiniana* in the Alps. The distribution is mapped according to Flora Alpina (Aeschimann & Lauber, 2004). The contact zone is mapped based on the records of intermediate herbarium specimens in Hörandl (1992). The map was created with *QGIS* (v.3.16.3). White boxes with text represent population IDs (see details in Table 1).

Two closely related European *Salix* species of section *Arbuscella* (as classified by Skvortsov, 1999), *S. foetida* Schleich. ex DC., and *S. waldsteiniana* Willd. were traditionally recognized as vicariant pair with a west–east distribution pattern (Aeschimann & Lauber, 2004; Hörandl, 1992; Rechinger, 1964; Skvortsov, 1999). Outside the Alps, *S. foetida* has a western distribution in the Pyrenees and Apennines, and *S. waldsteiniana* an eastern distribution in the Dinarid and Balkan Mountains. However, intermediate phenotypes have been observed in central regions of the Alps where their distributions overlap (Hörandl, 1992). Their sister position has been genetically confirmed by using phylogenomic data (Wagner et al., 2020), but a population genetic study on the sympatric zone is so far missing. Hence, it remained unclear whether these intermediate morphotypes would represent a hybrid zone, single F1 hybrids, or would just result from an overlap of morphological variation of the pure species (Figure 1). Moreover, the species tend to show differences in habitat preferences, with *S. waldsteiniana* primarily occurring on carbonate bedrock soils and *S. foetida* on silicate bedrock soils and moister habitats (Hörandl, 1992, Hörandl et al., 2012, Aeschimann & Lauber, 2004). Both species are diploid with 19 chromosome pairs (2 *n* = 38) (Büchler, 1985; Dobes et al., 1997; Neumann & Polatschek, 1972). The key morphological characteristics for identification are based on the anatomy of leaves and catkins. According to Rechinger (1964) and Hörandl et al. (2012), the two species can be identified by their leaf dentation patterns, with *S. foetida* having dense, pronounced, well‐perceptible teeth with well‐visible, brightly colored glands, and *S. waldsteiniana* having widely separated, less pronounced teeth with inconspicuous, darker glands. The leaves of *S. waldsteiniana* are also often larger, broadly elliptic to obovate compared to those of *S. foetida*, which are narrowly elliptic to lanceolate. Catkins, both male and female, are somewhat larger in *S. waldsteiniana*, but otherwise very similar in both species. The observed putative hybrid phenotypes, as described in Hörandl (1992), show intermediate dentation patterns with bright glands, resembling those of *S. foetida*, or they are densely dentated, but the glands are small and more similar in appearance to those of *S. waldsteiniana*. The size of the leaves was reported to be within the range of variability of both species. Observation of deformed and fruitless female catkins led to a hypothesis, that the intermediate individuals most likely represent reproductively unsuccessful first generation of hybrids. However, the study by Hörandl (1992) was based just on single herbarium specimens. To study morphological characters, accurate and objective measurements of population samples and statistical analyses (“morphometrics”) are required. Informative quantitative traits of floral or vegetative tissues can be measured, calculated, and compared as simple functions, usually as distance metrics (Lexer et al., 2009). Digital images of selected tissues are easily stored and can be analyzed using a set of various methodologies. These include the digital analysis of shapes, coined under the term “geometric morphometrics” (Mitteroecker & Gunz, 2009). Leaves offer several characteristics extensively used in traditional taxonomic keys for species identification (Cope et al., 2012).

The scope of this study was to investigate the putative hybrid zone of *S. foetida* and *S. waldsteiniana* in the European Alps using SNP molecular markers obtained via RAD‐seq and to conduct morphometric analysis of selected phenotypic leaf traits, based on population samples. With this, we aimed to answer the following questions: (i) Does hybridization occur between the two sister species? (ii) Is hybridization only local or is the hybrid zone larger than previously anticipated? (iii) What is the genetic structure of those hybrid zone populations and what are the evolutionary processes involved (e.g., hybrid speciation or permeable gene flow and introgression)? (iv) Can analyses of morphological traits unravel hybrid phenotypes, distinguish them from parental ones, and are they concordant with genetic structure among individuals/genetic ancestry of individuals?

## 2 MATERIALS AND METHODS

### 2.1 Sampling

In the years between 2014 and 2020, leaf samples of *S. foetida*, *S. waldsteiniana*, and putative hybrids were sampled over the species' distribution area in the European Alps. In total, 102 individuals were included in the study representing five populations of *S. foetida*, five populations of *S. waldsteiniana,* and two intermediate hybrid populations. For details, see Figure 1 and Table 1. Several undamaged, fresh leaves were removed from each selected individual and preserved in silica gel for DNA extraction. Herbarium vouchers for each sample were deposited in the herbarium of the University of Göttingen (GOET).

**TABLE 1:** Population sampling used in this study

Population ID	Country	Species (phenotype)	Locality	Latitude	Longitude	Altitude	No. of DNA samples used	No. of individuals used for morphometrics	Date of collection	Collector
CH3	Switzerland	*S. foetida*	Grand St. Bernard	45°53′45.1″N	7°11′11.9″E	2040	10	10	4.08.2020	Elvira Hörandl
CH11	Switzerland	*S. foetida*	Rhône Glacier	46°33′59.2″N	8°22′23.7″E	1780	3	0	18.7–19. 7. 2014	Susanne Gramlich
CH4	Switzerland	*S. foetida*	Julier pass, direction Tiefencastel	46°27′40.0″N	9°40′58.1″E	1905	10	10	26.07.2020	Elvira Hörandl
IT12	Italy	*S. foetida*	Vinschgau	46°31′33.8″N	10°35′10.9″N	1850	1	0	4.08.2015	Elvira Hörandl
IT6	Italy	Hybrid	Zirog Alps, Brennerbad	46°57′57.0″N	11°29′53.4″E	1855	11	11	2.08.2019	Elvira Hörandl
IT8	Italy	*S. waldsteiniana*	Seiseralm, Alpenrosenhütte	46°30′ 55.3″N	11°38′41.2″E	2035	11	11	8.08.2019	Elvira Hörandl
IT5	Italy	*S. foetida*	Seiseralm, Sonne	46°33′01.5″N	11°39′56.0″E	1827	11	11	8.08.2019	Elvira Hörandl
IT7	Italy	*S. waldsteiniana*	Grödnerjoch	46°32′ 46.8″N	11° 48′21.9″E	2050	11	11	6.08.2019	Elvira Hörandl
IT9	Italy	hybrid	Grödnerjoch, direction to Corvara	46°32′ 54.4″N	11°48′48.3″E	2056	11	11	6.08.2019	Elvira Hörandl
SI1	Slovenia	*S. waldsteiniana*	Srednji Golak	45°58′33″N	13°52′32″E	1383	10	10	13.06.2020	Pia Marinček
SI2	Slovenia	*S. waldsteiniana*	Ratitovec	46°14′17″N	14°5′56″E	1350	10	10	17.6. ‐ 18.6. 2020	Pia Marinček
AU10	Austria	*S. waldsteiniana*	Styria & Lower Austria	47°39′54.1″N–47°43′01.3″N	13°46′55.8″E–15°46′33.7″E	1550–1780	3	0	3.8. ‐ 8.8. 2017	Natascha Wagner & Katrin Scheufler

*Note*: The sampling was extended by two additional samples of *S. viminalis* published in Wagner et al. (2018) that were used as outgroup.

### 2.2 DNA extraction and RAD‐seq

Between 10 and 20 mg of silica‐dried leaf material per individual was extracted using the DNeasy Plant Mini kit (Qiagen, Hilden, Germany) following a modified manufacturer's protocol. The volume of AP1 buffer was adjusted between 400 and 450 μl, depending on the quantity of leaf material used in the extraction, the volume of RNase A was decreased to 2.5 μl, and incubation at 65°C was 30 min long. Incubation on ice was increased to a minimum of 30 min. The final elution was done using two times 50 μl of AE with an incubation time of 30 min at room temperature, each resulting in 100 μl DNA extract. The DNA quantity was measured with a Qubit 3.0 Fluorometer (Thermo Fisher Scientific, Waltham, USA). DNA extracts were sent to Floragenex Inc. (Beaverton, USA), for single‐end RAD‐seq library preparation using the *Pst*I restriction enzyme followed by sequencing on an Illumina HiSeq 2500 platform (Illumina Inc., San Diego, USA).

### 2.3 RAD‐loci assembly and SNP calling

After demultiplexing of 100 bp reads, the quality of sequences was checked using *FastQC* (v.0.11.9) (Andrews, 2010). Two software tools, *ipyrad* (Eaton & Overcast, 2020) and *STACKS* (v.2.2) (Catchen et al., 2013), were used to assemble the RAD‐loci, discover SNPs, and genotype the demultiplexed dataset. Because the assembly of homologous loci is dependent on the parameters set in a software tool, several test runs were performed to determine the best‐fitting settings for the threshold of similarity at which reads assemble into a homologous locus. For this, an empirical pipeline by McCartney‐Melstad et al. (2019) was used. For *ipyrad*, a clustering threshold of 93% was identified as the most appropriate. For *STACKS*, the de novo pipeline was used with the following parameters: the minimum number of perfectly matching raw reads (m) required to create a stack was set to 10, the maximum number of nucleotide mismatches (M) allowed between stacks within individuals to merge the stacks into one locus was set to 3, and finally, the maximum number of nucleotide mismatches (*n*) allowed between stacks between individuals to merge the stacks was set to 3. Further filters were applied to remove problematic aspects of the full RAD‐loci assembly, such as missing data, non‐informative loci, and linkage disequilibrium. In *ipyrad*, the parameter for minimum samples per locus was set to 81, which roughly corresponded to 80% of all individuals. An additional *ipyrad* assembly, with an inclusion of two outgroup *S. viminalis* samples (from Wagner et al., 2018), with the same settings, was constructed, adding to 104 individuals. In *STACKS*, a population map was prepared that divided the dataset into three groups. Two groups consisted of parental individuals outside the contact zone, and the third group consisted of all individuals within the contact zone, regardless of their phenotype. Only the loci present in 80% of the individuals within each group were kept (flags *r* and *p* set to 80 and 1, respectively). The amount of missing data was minimized by the use of *min‐maf* flag set to 0.05, where loci appearing less frequently than 5% were discarded. Potentially paralogous loci, putatively consequential to a recent salicoid duplication event (Tuskan et al., 2006), that are characterized by an excess of heterozygosity (Hohenlohe et al., 2011), were filtered based on their levels of heterozygosity and *F*_IS_ *‐*value. Loci above the observed heterozygosity of 60% were discarded with the *max‐obs‐het* flag, and loci with a *F*_IS_‐value below zero, calculated with the *fstats* flag, were added manually to a blacklist using the *sumstats* file and *Microsoft Office Excel* (v.2016) (Microsoft Corporation, Redmond, USA). Less informative parental loci (referring to the loci present in the populations of parental species outside the contact zone) that were out of Hardy–Weinberg equilibrium (HWE) at *p*‐value < 0.05 were extracted to a blacklist in the same manner (Gramlich et al., 2018). Since willows are dioecious (i.e., have separate male and female individuals), selfing is not possible and hence, high positive *F*‐values (inbreeding coefficients) are not to be expected. Because several analysis tools require unlinked markers, the *write‐single‐snp* flag was used where only the first SNP per locus was kept. To ensure the reproducibility of the results, the option of keeping the first SNP per locus was made. Next, the appropriate outputs (generated by the *plink* flag) were used in *PLINK* (v.1.90) (Purcell et al., 2007). The blacklisted loci were removed with the use of the *exclude* flag. To decrease missing data, SNPs with a genotyping rate below 10% were removed with the flag *geno*. This thorough filtering resulted in a discharge of several thousand initially assembled loci but ensured that the remaining dataset was appropriate for all the subsequent population genetics analyses.

### 2.4 Phylogenetic analysis and detection of reticulate evolution

To investigate the relationships between the individuals, two tree‐building methods were applied. A maximum‐likelihood (ML) building approach with a GTR + Γ model of nucleotide substitution was used in the software *RAxML* (v.8.2.4) (Stamatakis, 2014) with rapid bootstrapping analysis of 100 replicates. The ML tree was generated using the complete RAD‐loci alignment of the *ipyrad* assembly with outgroups. It was visualized with *FigTree* (v.1.4.4) (Rambaut & Drummond, 2012). The branch support values of the ML tree were quantified with the Quartet Sampling (QS) method (Pease et al., 2018), implemented in *quartetsampling* (v.1.3.1). Phylogenetic networks extend phylogenetic trees and can more explicitly represent the reticulate evolutionary history of taxa in processes like hybridization (Huson & Bryant, 2006). Neighbor net (NN) (Bryant & Moulton, 2004) was done using *SplitsTree* (v.4.17.1) (Huson & Bryant, 2006) with 100 bootstrap replicates on the complete SNP alignment of the *ipyrad* assembly with outgroups. The *HyDe* software package (Blischak et al., 2018) uses a phylogenetic inference approach to test the dataset for hybridization patterns. Estimated *γ*‐values explain detected hybridization patterns: values between 0.4 and 0.6 hint to (recent) hybridization events, and values between 0.2–0.4 and 0.6–0.8 hint to introgression or ancient hybridization events. The data can be tested for populations or individuals as specified in a population map. For this analysis, the complete RAD‐loci alignment of the *ipyrad* assembly with outgroups was used. The population map defined all parental individuals from populations outside the contact zone as either *S. waldsteiniana* or *S. foetida* population, and each hybrid individual was assigned its own group. A file of 55 triplets was created to test each individual within the contact zone as a hybrid of both parental species, where *S. foetida* population was assigned as the first parent and *S. waldsteiniana* as the second parent. Triplets with significant hybridization (*p*‐value < 0.05) were tested with 100 bootstrap replicates, using *bootstrap_hyde.py* script. The average *γ*‐values of the replicates were visualized with *ggplot2* (v.3.3.5) package (Wickham, 2016) in *R* (v. 4.1.2) (R Core Team, 2021).

### 2.5 Population genetics

Several population genetics analyses were performed on the *STACKS* assembly. To explore the presence of distinct genetic groups within the dataset, an *sNMF* analysis was performed with the function *snmf* in *R* package *LEA* (v.1.4.0) (Frichot & François, 2015), where ancestry proportions of *K* ancestral populations are estimated with least‐square estimates. Initial runs identified a biased grouping of population IT5, presumably as a result of high inbreeding or clonality of these individuals. The population was spatially very restricted and surrounded by ski slopes (field obs. E. Hörandl), and has probably undergone a recent drastic size reduction and genetic bottleneck. To avoid bias due to strong departure from HWE (Hedrick, 2011) and overestimates of *K's* for structure analyses (Frichot et al., 2014), the dataset was reduced to 94 specimens, removing all but three IT5 individuals. As an additional comparison to the *sNMF* method, *STRUCTURE* (v.2.3.4; Pritchard et al., 2010) was run on the *STACKS* assembly for the K range 1–13 with 50,000 MCMC iterations and a 10,000 burn‐in period and on 46,695 unfiltered, unlinked SNPs of the *ipyrad* assembly with *K* ranging from 1 to 20. The most likely *K* was determined with *STRUCTURE HARVESTER* (v.0.6.94) (Earl & vonHoldt, 2012) using Evanno's method (Evanno et al., 2005). The separate runs per *K* were summarized in a single output using *CLUMPP* (v.1.1.2) (Jakobsson & Rosenberg, 2007), with a *Greedy* method for 10,000 repeats. A higher‐resolution genetic structure analysis was performed with the *RADpainter* and *fineRADstructure* pipeline (Malinsky et al., 2018), where co‐ancestry is calculated based on haplotype linkage information. The heatmap was generated with *R*. To assign the parental and hybrid classes, software *NewHybrids* (v.1.1 beta) (Anderson & Thompson, 2002) was run for 100,000 MCMC iterations with a 25,000 burn‐in period on the reduced dataset of 94 individuals and restricted for the first 300 loci due to the software's computational constrains. Divergence of the populations was measured via statistical evaluation of genetic variance using the *F*_ST_‐values calculated with the *R* package *genepop* (v.1.1.7) (Raymond, 1995). Here, all parental individuals were concatenated into two parental populations, and each contact zone population was treated as a separate group to evaluate pairwise divergence between each of the parental species, as well as other contact zone populations. With the calculation of the isolation‐by‐distance (IBD) slope between the geographic and genetic distance matrices, using the *adegenet* (v.2.0.1) (Jombart, 2008) *R* package, the pattern of spatial genetic variation was investigated.

### 2.6 Morphometrics

Morphometric measurements were applied to the 95 herbarium vouchers of the collected specimens. To represent an individual's average leaf, up to 10 mature leaves per herbarium specimen were selected. Each individual leaf was positioned on a blank paper sheet with a 10 × 10 mm black square as a scale, and scanned with a Canon CanoScan LiDE 220 (Canon, Tokyo, Japan) scanner, producing a high‐quality image. The number of teeth on the perimeter of each leaf was counted manually, and parameters length, width, widest point (distance from the base of the leaf to the widest part), perimeter, and area were measured on the scanned images with *Digimizer* (v.5.7.2) image analysis software (MedCalc Software Ltd, Ostend, Belgium). The ratios between length and width, as well as length and widest point, were calculated to quantitatively represent the shape of each leaf. A higher length‐to‐widest point ratio describes an ovate or lanceolate (in combination with a high length‐to‐width ratio) leaf shape and a lower ratio describes an obovate leaf shape. In total, it was possible to obtain 781 leaves of sufficient quality for morphometrics, adding up to approximately eight leaves per individual. A multivariate analysis approach was used to investigate several morphometric variables in a single analysis. Based on the correlation inspection performed in *R* with the *cor* function, four independent metrics (leaf area, no. of teeth per cm, length‐to‐width ratio, length‐to‐widest point ratio) were selected for the multivariate principal component analysis (PCA), which was performed with the function *prcomp* and visualized with *ggbiplot* (v.0.55) (Vu, 2011) in *R*. Discriminant analysis of principal components (DAPC) (Jombart et al., 2010) in the package *adegenet* was used as an evaluation of the groups of individuals based on both phenotypical identification and identification of genetic groups (pure or admixed), as assigned in the *sNMF* analysis for *K* = 2.

## 3 RESULTS

### 3.1 RAD‐seq results

On average, 6.94 million reads were assembled per individual. No population significantly deviated from the mean. The *ipyrad* run on 102 samples generated 47,297 RAD‐loci with 315,576 SNPs with 6.3% of missing data. The final *ipyrad* assembly including outgroup consisted of 47,899 RAD‐loci with 336,649 SNPs and 6.6% missing SNP data. The final *STACKS* assembly generated 50,924 RAD‐loci with 329,518 SNPs, comparable to the outcome of the *ipyrad* assembly. The final STACKS assembly with unlinked SNPs used for most population genetic analyses consisted of 6173 SNPs with 4.4% missing SNP data.

### 3.2 Phylogenetic network and reticulate evolution

**FIGURE 2:**
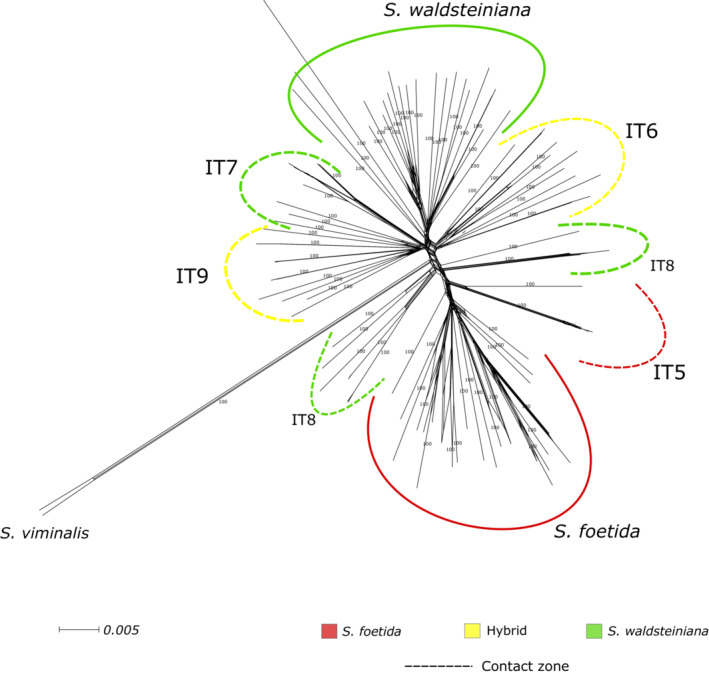
Neighbor‐net network of 104 individuals, including the two outgroup *S. viminalis* individuals with displayed bootstrap values for longest branches. The scale for branch length is indicated on the left side below. Individuals are colored by populations and marked with corresponding population identifiers (Table 1). Colors indicate phenotype as described in the legend. Dotted lines represent populations collected in the contact zone.

The topology of the NN phylogenetic network was generally well supported with high bootstrap values (Figure 2). All locally sampled populations, except for a contact zone population IT8 (identified as *S. waldsteiniana*), formed well‐supported clusters. The populations of *S. foetida* and *S. waldsteiniana* sampled outside the contact zone were positioned farthest apart, indicating the two most diverging monophyletic clades, with the remaining contact zone populations forming paraphyletic groups positioned intermediately to both parental clades. Some populations seemingly consisted of several highly related individuals, with this pattern being the most prevalent in the population IT5, where all individuals but one showed high genetic similarity. The topology of the ML tree (Appendix S1) was similar to the NN phylogenetic network. Quartet sampling analysis revealed discordances of the topologies of the contact zone populations IT6 and IT8, and the clade conjoining populations IT7 and IT9, as well as population AU10. The individuals from this population formed long branches in the NN network.

**FIGURE 3:**
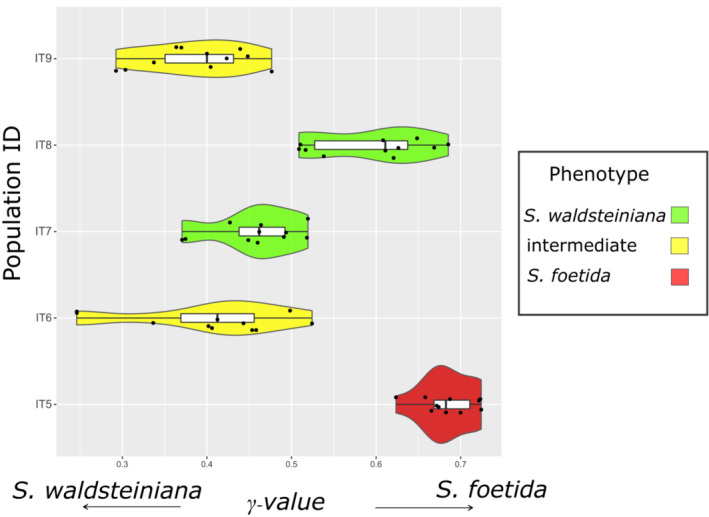
Calculated *γ*‐values from *HyDe* analysis for each of the 55 individuals of the five populations from the contact zone, represented with violin plots. Higher values indicate higher similarity to *S. foetida*, and lower values indicate higher similarity to *S. waldsteiniana* genetic patterns. Populations are labeled with corresponding population identifiers (Table 1). Colors represent observed phenotype, as described in the legend on the right.

In the *HyDe* analysis, significant hybridization between the “parental” *S. foetida* and *S. waldsteiniana* populations was detected for all 55 individuals of the putative hybrid zone (Figure 3). Population IT5, identified as *S. foetida*, showed the highest portions of the *S. foetida* genetic patterns (*γ*‐values 0.62–0.72). Population IT8, identified as *S. waldsteiniana*, also showed high proportions of the *S. foetida* genetic patterns (*γ*‐values 0.51–0.69), however, several individuals showed a nearly equal admixture of both parental patterns. Population IT7, identified as *S. waldsteiniana*, displayed the most equally admixed patterns of both parents (*γ*‐values 0.37–0.52). Populations IT6 and IT9, both identified as hybrids, showed the highest variance (*γ*‐values 0.24–0.52 and 0.29–0.48, respectively), with admixture patterns slightly shifted toward *S. waldsteiniana*.

### 3.3 Population genetics

**FIGURE 4:**
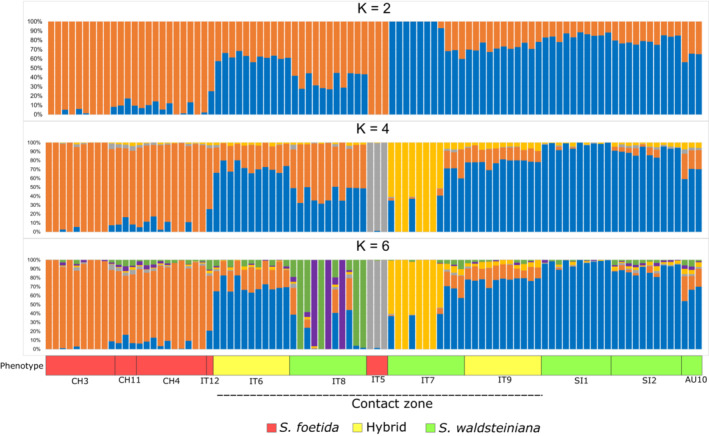
Results of the *sMNF* analysis for *K =* 2, 4, and 6, for the reduced dataset of 94 individuals. Results are represented with stacked bar plots. Different colors of the plots represent different genetic partitions, with their posterior probabilities in the y‐axis as calculated in the analysis. Populations are ordered by their geographical location from west to east, labeled by their identifiers (Table 1), and colored by phenotype as described in the legend below. The dotted line marks populations from the contact zone.

The plot of calculated entropy suggested that the plateau was reached around *K* = 6 and therefore *K* = 2, 4, and 6 were used (Figure 4). For *K* = 2, the observed genetic patterns of admixture matched those calculated in the *sNMF* analysis of the *ipyrad* assembly (Appendix S2) and those calculated in *STRUCTURE* (Appendix S3). However, both parental populations seemingly showed higher portions of the other species' gene pool in the *sNMF* analysis results. This is more pronounced in *S. waldsteiniana*, where individuals from populations SI1 and SI2 showed between 11.5% and 25.0% posterior probability of the *S. foetida* genetic patterns (mean = 17.8%). The three individuals collapsed into the AU10 population showed even higher admixture, with posterior probabilities for *S. foetida* genetic cluster ranging between 34.5% and 43.6% (mean = 37.8%). Comparably, several *S. foetida* individuals from outside the contact zone showed no *S. waldsteiniana* genetic patterns, and the highest observed posterior probability was 25.1% (mean = 6.2%). With higher values of *K*, internal structures of local populations were recognized as genetic patterns. Decreased variation in posterior probabilities of different genetic groups could be observed in *S. waldsteiniana* populations outside the contact zone when *K* was 4 and 6 (the exception was population AU10). On the contrary, the posterior probabilities of other genetic groups increased in *S. foetida* individuals outside the contact zone with an increase in *K*.

**FIGURE 5:**
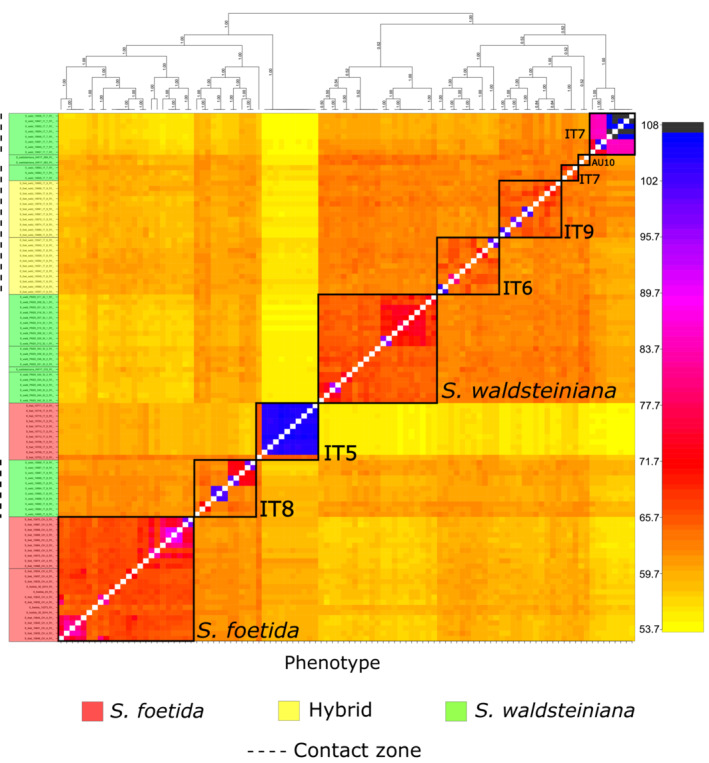
Results of the *RADpainter* and *fineRADstructure* analyses represented in a heatmap plot of the calculated coancestry coefficient matrix. The scale is on the right (black: highest coancestry, yellow: lowest coancestry). On the left, individuals are colored by their identified phenotype as explained in the legend below. The dotted line marks populations located in the contact zone. Black squares on the coancestry matrix mark samples from the same population and are labeled accordingly (Table 1). The populations of *S. waldsteiniana* (with the exception of two samples from population AU10) and *S. foetida* from outside contact zones are collapsed into two large populations and labeled accordingly.

The analysis of the ancestry covariance matrix with *RADpainter* (Figure 5) showed high coancestry coefficients between all individuals of the *S. foetida* and *S. waldsteiniana* populations (dark orange colors). The only exceptions were two *S. waldsteiniana* individuals from population AU10 that were nested in one of the paraphyletic groups of the contact zone populations. For some populations, clear internal structuring with high coancestry coefficients (red colors, Figure 5) could be observed. Populations IT5 and IT7 showed high portions of individuals with extremely high coancestry coefficients (blue and black colors), while in other groups, single pairs of individuals exhibited such patterns. Two larger structural groups with higher coancestry coefficients could be observed, one including all *S. foetida* individuals, and populations IT8 and IT5 from the contact zone, and the second group including all *S. waldsteiniana* individuals, and populations IT6, IT9, and IT7 from the contact zone.

The *NewHybrids* analysis was able to assign most of the putative parental individuals either to the first parent (*S. foetida*) or to the second parent (*S. waldsteiniana*) with high posterior probabilities and could identify several individuals from the contact zone as hybrid progeny (Appendix S4). These were classified as either F2 hybrids or backcrosses to *S. waldsteiniana*. Additionally, two of the individuals of the population AU10, sampled outside the contact zone, were recognized as admixed.

The *F*_ST_‐values calculated among tested populations ranged between 0.06 and 0.43 (Table 2). *Salix foetida* and *S. waldsteiniana* populations did not show the highest pairwise *F*_ST_‐value among all groups (0.16). The highest *F*_ST_‐values were observed between population IT5, identified as *S. foetida* (0.31–0.43), and all other populations. Of that, the lowest (0.31) was the *F*_ST_‐value with the parental *S. foetida* population and the highest (0.43) with population IT7, identified as *S. waldsteiniana*. Of all identified *S. waldsteiniana* populations, population IT7 was the most diverged to parental *S. foetida*, showing the highest *F*_ST_‐value (0.22), followed by the parental *S. waldsteiniana* population (0.16). Populations IT8, identified as *S. waldsteiniana*, showed similar *F*_ST_‐values with both parental populations (*S. foetida* [0.10], *S. waldsteiniana* [0.11]). Populations IT6, IT9, and IT7 had lower *F*_ST_‐values with parental *S. waldsteiniana* (0.060, 0.063, and 0.15, respectively) than with the parental *S. foetida* population (0.12, 0.15, and 0.22, respectively). The IBD analysis showed insignificant spatial correlation of the genetic and geographic distances, hinting at a stronger prevalence of genetic structuring that is not influenced by spatial segregation.

**TABLE 2:** Matrix of calculated pairwise *F*_ST_‐values between examined populations

Phenotype	Population ID	*S. foetida*	IT5	IT6	IT9	IT7	IT8	*S. waldsteiniana*
*S. foetida*	*S. foetida*	0	0.31	0.13	0.15	0.22	0.10	0.16
*S. foetida*	IT5	0.31	0	0.35	0.37	0.43	0.34	0.35
Hybrid	IT6	0.13	0.35	0	0.07	0.16	0.09	0.06
Hybrid	IT9	0.15	0.37	0.07	0	0.15	0.11	0.06
*S. waldsteiniana*	IT7	0.22	0.43	0.16	0.15	0	0.19	0.15
*S. waldsteiniana*	IT8	0.10	0.34	0.09	0.11	0.19	0	0.11
*S. waldsteiniana*	*S. waldsteiniana*	0.16	0.35	0.06	0.06	0.15	0.11	0

*Note*: Populations outside the contact zone were collapsed into *S. waldsteiniana* (populations SI1, SI2, and AU10) or *S. foetida* (populations CH3, CH4, CH11, and IT12) population.

### 3.4 Morphometrics

**FIGURE 6:**
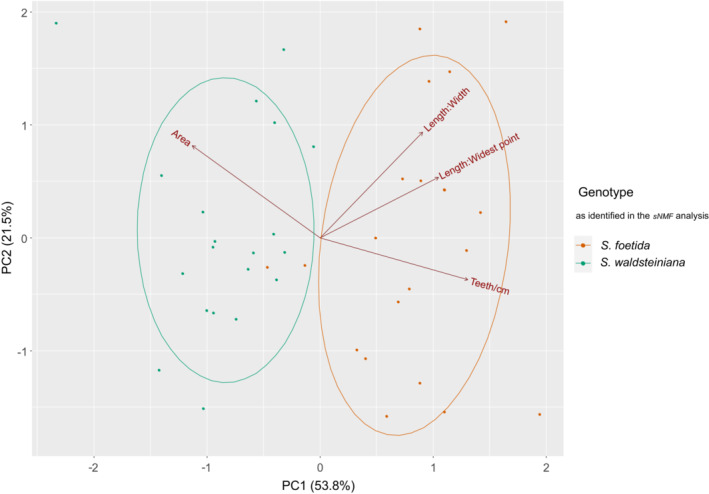
Plot of the first two principal components for the PCA of morphometric data of all parental *S. foetida* (populations CH3 and CH4) and *S. waldsteiniana* (populations SI1 and SI2) individuals with 95% confidence ellipses.

Multivariate PCA of morphometric traits, averaged by the individual, revealed that the two groups of parental *S. foetida* and *S. waldsteiniana* individuals outside the contact zone showed separation on the axis of the first principal component (See Figure 6). PC1 described 53.8% of the total variation and PC2 21.5%. PGMPs calculated in DAPC were 0.95 for *S. foetida*, 1.0 for *S. waldsteiniana*, and 0.975 for the correct assignment of a random individual.

**FIGURE 7:**
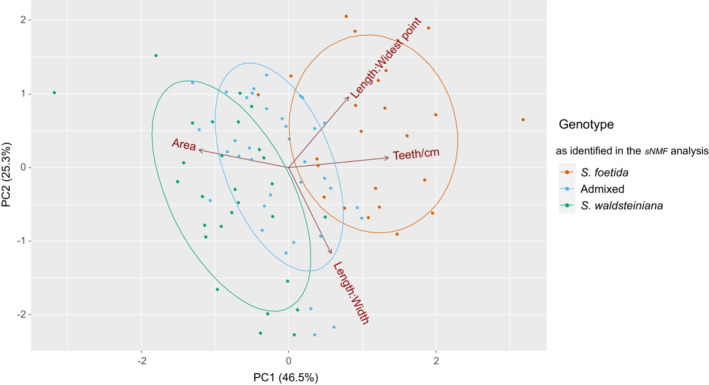
Plot of the first two principal components for the PCA of 87 morphometrically assessed individuals (population IT5 reduced to three individuals) with 95% confidence ellipses. The groups assigned were based on the rate of admixture as calculated in the *sNMF* analysis.

When PCA was applied to the reduced dataset of 87 individuals that were investigated in the *sNMF* analysis, the calculated PC1 (46.5%) and PC2 (25.3%) were only slightly different (Figure 7). The two parental groups did not show an overlap of the 95% confidence ellipses, and individuals that were recognized as admixed were positioned intermediately between the parental clusters with a slight skew toward the *S. waldsteiniana* parental cluster. Calculated PGMPs were 0.74 for *S. foetida*, 0.86 for *S. waldsteiniana*, 0.89 for admixed individuals, and 0.84 for the correct assignment of a random individual.

## 4 DISCUSSION

### 4.1 Hybrid zone formation of *S. foetida* and *S. waldsteiniana*

Secondary contact hybridization is predicted as a frequent outcome of range fluctuations of plant species due to climatic changes in the European Alps (Hewitt, 2004, 2011). With the use of RAD‐seq technology, it was possible to give evidence for the existence of a homoploid hybrid zone of the sister species *S. foetida* and *S. waldsteiniana* within their sympatric distribution area in the Alps. Other than expected, genetic structure suggests an evolution of hybrids beyond local F1 hybrids. The morphological patterns clearly separated the parental species but revealed intermediate or parental phenotypes for the hybrid populations.

### 4.2 Reticulate relationships of hybrid zone populations

The phylogenetic analysis and reconstruction of the ML tree and NN phylogenetic network revealed the division of the two species into clear monophyletic clusters, whereas populations sampled in the contact zone formed paraphyletic groups (Figure 1, Appendix S1). The presence of conflicting topologies, as a result of reticulate networks, was suggested by calculated *QS* scores for all clades of the contact zone populations (Appendix S1). A discrepancy between phenotypic identification and phylogenetic topology could be observed. For example, population IT8, with identified *S. waldsteiniana* phenotype, showed greater genetic similarity with *S. foetida* populations than *S. waldsteiniana* populations in the phylogenetic studies. An analysis of reticulate evolution with *HyDe* suggested that all populations from the contact zone resulted from admixture of both species (Figure 3), hinting at patterns of recent hybridization (in IT7, IT9, and partially in IT8 and IT6) and introgression (in IT5, IT8, and partially in IT6 and IT9). Calculated *F*_ST_‐values between all parental populations outside the contact zone were low, indicating little genetic divergence between the taxa, but were still higher than in pairwise comparisons of admixed populations IT6, IT9, and IT8 (Figure 3, Table 2). The general patterns observed in both phylogenetic inference and population genetics analysis indicate the presence of a broad hybrid zone of *S. foetida* and *S. waldsteiniana* in the Alps, which is most likely linked to post‐glacial recolonization of the area, following the last glacial maximum (Hewitt, 2004, 2011). This is in accordance with several plant taxa that were found to hybridize in the Alps (e.g., *Saxifraga* in Gugerli, 1997, *Euphrasia* in Liebst, 2008, and *Tephroseris* in Pflugbeil et al., 2021).

Phylogenetic approaches provided a good overview of the reticulate relationships of the examined populations. However, the process of incomplete lineage sorting (ILS) may result in the same patterns and therefore cannot be excluded from the interpretation (Galtier & Daubin, 2008). The method implemented in *HyDe* tries to overcome ILS by testing for specific hybridization hypotheses (Elworth et al., 2019) and can be interpreted more confidently as signal of true hybridization. The phylogenetic invariant approach used in this software first models a coalescent tree along the tested parents, which accounts for ILS, and then tests for hybridization with *D‐*statistics on coalescence‐independent sites (Kubatko & Chifman, 2019). However, the SNPs across alleles might be non‐randomly associated through processes of linkage and selection, resulting in increased levels of linkage disequilibrium (Flint‐Garcia & Thornsberry, 2003), which could affect the observed patterns. Therefore, population genetics approaches are better suited to identify true hybridization patterns. In the filtered and unlinked SNP assembly of *STACKS*, these effects were most likely reduced. The results of these analyses showed a clear admixture of parental genomes in the contact zone. The use of both datasets in this study acts reciprocally and supports the interpretation.

### 4.3 Genetic structure of hybrid populations

The two sister species could be referred to separate genetic clusters in the population genetics analyses, however, even individuals outside the contact zone showed some degree of admixture. Low *F*_ST_‐values between *S. waldsteiniana* and *S. foetida* populations outside the contact zone were an additional indicator of the low genetic divergence of both species. Intraspecific diversity of molecular characters may indicate historic hybridization and introgression of the two species, which has been documented in other *Salix* species (Hardig et al., 2000), as well as other woody plant genera (e.g., *Alnus glutinosa* in King & Ferris, 1998, *Quercus* in Petit et al., 1997), using plastid DNA markers. Wagner et al. (2018) attributed the same processes to the observed topology in the *Chamaetia/Vetrix* clade, after a phylogeny was inferred from the RAD‐seq dataset. *S. foetida* and *S. waldsteiniana* are sister species within this clade. The whole section seems to have a largely shared gene pool, resulting in low divergence across the whole clade, and hybridization is possible across different sections, not only within closely related species pairs (Gramlich et al., 2018; Wagner et al., 2018). In the hybrid zone of the distantly related species *S. helvetica* and *S. purpurea*, the calculated *F*_ST_‐value between the two species was contrastingly higher (*F*_ST_‐value *=* 0.53 for filtered loci in Gramlich et al., 2018) than for *S. foetida* and *S. waldsteiniana* in this study (*F*_ST_‐value *=* 0.16 for filtered loci). The genetic structure of populations showed that parental *S. waldsteiniana* individuals contain higher portions of *S. foetida* genetic patterns, which could be due to limited population sampling of the former in the northeastern range outside the contact zone. However, it could be also hypothesized that gene flow between these two species has historically been more directed toward *S. waldsteiniana*. The process might have been mitigated by a positive selection of ecologically favorable alleles that were incorporated into the genome through admixture, as was observed in other plant genera (e.g., *Helianthus* in Rieseberg et al., 2003, *Iris* in Arnold et al., 2012). Indeed *S. waldsteiniana* seems to tolerate a wider spectrum of ecological niches. It can grow in drier habitats and is not strictly bound to carbonate bedrock soils, whereas *S. foetida* seems to inhabit a much narrower spectrum of habitats with sufficient moisture and strictly silicate bedrock substrate (Aeschimann & Lauber, 2004; Hörandl, 1992). It might be possible that adaptive alleles of *S. waldsteiniana*, allowing a broader tolerance of habitat conditions, were obtained through ancient introgression.

Different degrees of genetic admixture in consistent patterns could be observed in populations of the contact zone throughout all population genetics analyses. The observed gradient of divergence does not seem to follow a geographic differentiation pattern according to the isolation‐by‐distance analysis but is characterized by locally established genetic patterns of admixture as a result of hybridization. Thus, the results did not support the hypothesis by Hörandl (1992) that populations with intermediate individuals solely consist of a continuously formed F1 generation. Even though catkin deformations and lack of seeds in mature fruits hinted to lower fertility rates of intermediate forms (Hörandl, 1992), our *NewHybrids* analysis suggests that the natural hybrids of *S. foetida* and *S. waldsteiniana* do form populations and are capable of reproduction beyond the first hybrid generation by producing backcross and F2 hybrids genotypes. This is often the case for naturally hybridizing plant taxa (Arnold, 1997, 2006; Mallet, 2005), and was frequently observed in *Salix* as well (Fogelqvist et al., 2015; Gramlich et al., 2018; Hardig et al., 2000). Reproductive isolation of hybrids from the parental species via genetic barriers is less likely than ecological isolation (Mallet, 2005, 2007; Yakimowski & Rieseberg, 2014). The apparent absence of genetic incompatibilities seems to be especially true for genus *Salix* in general, but the roles of ecological factors as crossing barriers are poorly explored for alpine willows (Wagner et al., 2021). The prevalence of admixed genotypes in the hybrid zone populations could be a sign of higher relative fitness of hybrids in instances where they occupy the same habitats as their parents (Arnold & Martin, 2010). However, we did not collect fitness data for the populations studied here; the two species are differentiated in habitat preferences in their allopatric ranges (*S. foetida* on more acid, wetter soils than *S. waldsteiniana*, but with an overlapping range; Aeschimann & Lauber, 2004). Population IT8 showed the most equal rates of both parental genetic clusters (Figures 4 and 5) and relatively equal convergence with both parental populations (Table 2). In the *NewHybrids* analysis, the allelic frequencies within this population were recognized as strong F2 hybrid generation signals (Appendix S4) with high posterior probabilities, except in one individual that exhibited a substantial portion of *S. waldsteiniana* backcross frequency patterns. It seems that this population might represent a locally established hybrid lineage with the potential for hybrid speciation (Hörandl, 2022). Since other hybrid zone populations of the two analyzed species in this study show admixture with one of the parents, reproductive isolation in this population is more likely linked to ecological isolation of a hybrid lineage. Specifically, in the complex geological environment of the Dolomites in South Tyrol (Figure 1), different bedrock conditions are available on small scales and would foster local differentiation of populations. Patchy ecological conditions would support the formation of mosaic hybrid zones as observed in other willows (Gramlich & Hörandl, 2016). Other *Salix* hybrids were observed to exhibit higher tolerances to nutrient deficiency and soil acidity than either of the parental species (Gramlich et al., 2016), which could enable them to inhabit habitats that were less suitable for both parents. Locally isolated homoploid hybrid populations leading to hybrid speciation could be observed in Brochmann et al. (2000) where an endemic plant species *Argyranthemum sundingii* was observed to have originated from hybridization of two species followed by spatial separation. Adaptive divergence of lineages also plays an important role in processes of speciation, as exemplified in *Iris nelsonii*, a species of homoploid hybrid origin that is ecologically divergent from its progenitors (Taylor et al., 2011).

Population IT5 (the remaining three individuals) and the majority of the population IT7 were seemingly classified as parental species' clusters with no (or only little) admixture with other populations in the *sNMF*, as well as in the *STRUCTURE* analysis for *K* = 2 (Figure 4, Appendices S2 and S3). High *F*_ST_‐values (Table 2) between these populations and parental populations showed a seemingly high divergence. Surprisingly, two *S. waldsteiniana* individuals from the population AU10, sampled outside the contact zone, showed patterns of introgression in genetic structure analyses and were classified as F2 hybrids in the *NewHybrids* analysis with high posterior probabilities (Appendix S4). It is possible that treating these single individuals as a part of a larger population throughout the filtering steps of *STACKS* assembly for population genetics analyses, presented a bias, as investigated by Graham et al. (2020). More population samples outside the contact zone would be needed to clarify population structure.

Within the analyzed area, no IBD could be confirmed. Willows are capable of long‐distance dispersal of both pollen and seeds, so it is possible that gene flow acts over large geographical scales. Possibly, the two species first came into contact in the central Alps during the latest post‐glacial recolonization of the area or earlier interglacial periods of the Pleistocene. Not much is currently known about the biogeographical history of *Salix* in the Alps (Wagner et al., 2021). For *S. waldsteiniana*, a recolonization from the southeastern refugia of the Alps (Merxmüller, 1952) or from the Balkan Mountains is likely according to present distribution patterns. These results call for a more complete sampling of *S. waldsteiniana* populations in its eastern expansion range border in the Alps that would be needed to explain the observed patterns of admixture.

### 4.4 Comparison of morphological traits and molecular data

Parental phenotypes, inferred from individuals outside the contact zone, could clearly be distinguished in the PCA analysis of the selected leaf traits. High PGMP confirmed the delimitation into two groups and phenotypic separation of two species based on the four examined leaf trait parameters. Leaf area and dentation density contributed most to interspecific variability, followed by length‐to‐widest point ratio and length‐to‐width ratio measurements (Figure 6). The results of the analysis are consistent with the current identification characters used in Hörandl et al. (2012) and support the current taxonomic treatment. In willows, morphological variability within species strongly depends on habitat conditions (Neumann, 1981), and often overlaps even between genetically distinct species (Hardig et al., 2000; Triest, 2001). Habitat conditions in willows may influence leaf size, leaf surface (rugose vs. flat), and density of indumentum of leaves, whereas leaf margins and dentation are usually highly stable and provide species‐specific characters (Neumann, 1981). The dentation of leaf margin and leaf shape (length/width ratios) are the strongest discriminant features of *S. foetida* and *S. waldsteiniana*. Both species are used as ornamentals in small gardens and keep their characteristic features in cultivation (Newsholme, 1992). Hence, we suppose that the distinct phenotypic traits in the parental species are heritable and not directly influenced by variable habitat conditions. In our case, morphological differentiation between parental species appears to be quite distinct, and intermediacy is confirmed as a result of hybridization and not just overlapping variation.

The distinction of the two species was still possible when all populations from the contact zone were included and assigned a group based on the phenotypic identification in the field. Individuals identified as *S. foetida* and *S. waldsteiniana* showed an overlap, as was also evident from the calculated average PGMP, which was lower for both species. These results suggest that phenotypes associated with each of the species are more similar and intermediate within the contact zone, than outside of it. Phenotypically identified hybrids nevertheless show the most intermediate characters and overlap with both parental groups. However, the genetic analyses showed that morphologically identified phenotype was not always congruent with genetic data. Genetic identification of parents and hybrid classes, as defined by the results of the *NewHybrids* analysis, resulted in a lower overlap of the parental clusters in the PCA analysis. The reduction in population IT5, the only contact zone population identified as *S. foetida,* might contribute to a lesser overlap as well. The F2 hybrid cluster and *S. waldsteiniana* backcross cluster show a nearly complete overlap, which suggests that individuals with different ratios of admixture cannot be phenotypically differentiated. Beside introgression, also segregation of characters in F2 and later hybrid generations can result in incongruence of molecular and morphological data (e.g., Hodač et al., 2018). Hence, the morphotypes in such hybrid zones do not necessarily reflect the genetic constitution of individuals.

Thompson et al. (2021) proposed that in hybridizing populations, individuals that are phenotypically similar to one parent should have a relatively high fitness and that phenotypic combinations of mismatches of parental characters are more detrimental. Congruent in this study is the observation of parental bias, but the multivariate approach does not allow for the investigation of single individual morphometric traits to search for combinations of mismatches. Instead, the multivariate PCA approach offers a more concise picture where intermediacy and parent bias can be assessed through a combination of all tested characters. The study of *S. eriocephala* and *S. sericea* hybrid zone by Hardig et al. (2000) has made very similar observations compared to our study. The analyzed morphometric traits showed either intermediacy or were more similar to parental characteristics. Several phenotypically identified *S. eriocephala* individuals were found to be genetically admixed. If homoploid *Salix* hybridization events frequently result in parental‐like phenotypes, as seen in this study and Hardig et al. (2000), it can be assumed that willow hybrid zones might be broader than currently estimated, which calls for further studies with a more extensive sampling throughout the entire range of the contact zone, and not solely focused on areas of putatively intermediate forms. The results of the morphometric studies might also be biased by the limited selection of morphometric traits (Lexer et al., 2009). However, the lack of morphometrically feasible traits is common in genus *Salix* (e.g., six traits in Hardig et al., 2000), mainly because of the scarcity of floral characters (Hörandl, 1992). In a hybrid zone study of *S. eriocephala* and *S. sericea*, measuring the content of chemical compounds was proven to be more concordant with genetic classification, than morphometry (Hardig et al., 2000). Perhaps the investigation of secondary metabolites is more informative in disentangling relationships within the genus *Salix* (Nyman & Jukunen‐Tiitto, 2005) and to identify hybrid individuals (Hardig et al., 2000; Oberprieler et al., 2013) than morphological analyses, which are restricted to very few and very variable characters, as exemplified in this study and other studies on willows (Hardig et al., 2000; Wagner et al., 2018; Wu et al., 2015). Finally, ecological surveys of the investigated populations might provide additional insight into relationships between phenotypes and ecological conditions of parental and hybrid habitats. Mosaic‐like habitat conditions, especially in geologically diverse parts of the Alps, likely increase the chances of co‐occurrence and hybridization of the species, which needs to be studied in the context of ecological niches of the species in the whole distribution area.

## 5 CONCLUSION

Our study detected a homoploid hybrid zone between two sister species of willows in the European Alps by using population genomics and a morphometric approach. The hybrid zone resulted probably from secondary contact hybridization due to past climatic changes, and spans over the sympatric area of the two species in the eastern Alps. The hybrid populations evolved via introgression and lineage formation beyond the F1 generation, and express intermediate or parental phenotypes. The formation of this geographically large hybrid zone might have been aided by low genetic divergence of parental species, the ability of long‐distance dispersal by both pollen and seeds, and incomplete habitat differentiation.

## AUTHOR CONTRIBUTIONS

**Pia Marincek:** Formal analysis (lead); investigation (lead); methodology (lead); visualization (lead); writing – original draft (lead); writing – review and editing (supporting). **Loic Pittet:** Formal analysis (supporting); investigation (supporting); writing – review and editing (supporting). **Natascha Dorothea Wagner:** Conceptualization (supporting); formal analysis (supporting); investigation (supporting); resources (supporting); supervision (supporting); writing – original draft (supporting); writing – review and editing (supporting). **Elvira Hörandl:** Conceptualization (lead); funding acquisition (lead); project administration (lead); resources (lead); writing – review and editing (lead).

## Funding information

Open Access funding enabled and organized by Projekt DEAL.

## CONFLICT OF INTEREST

None declared.

## Supporting information


Appendix S1.

Appendix S2.

Appendix S3.

Appendix S4.

## Data Availability

All demultiplexed raw read data resulting from RAD‐seq were submitted to the National Center for Biotechnology Information (NCBI) in the SequenceReadArchive (SRA) under the BioProject ID PRJNA873074.
