# Supplementary figures and images for: Evolution of a hybrid zone of two willow species (Salix L.) in the European Alps analyzed by RAD‐seq and morphometrics
Source: Ecol Evol. 2023 Jan 4;13(1):e9700. doi: 10.1002/ece3.9700 (PMC9811612; doi:10.1002/ece3.9700)

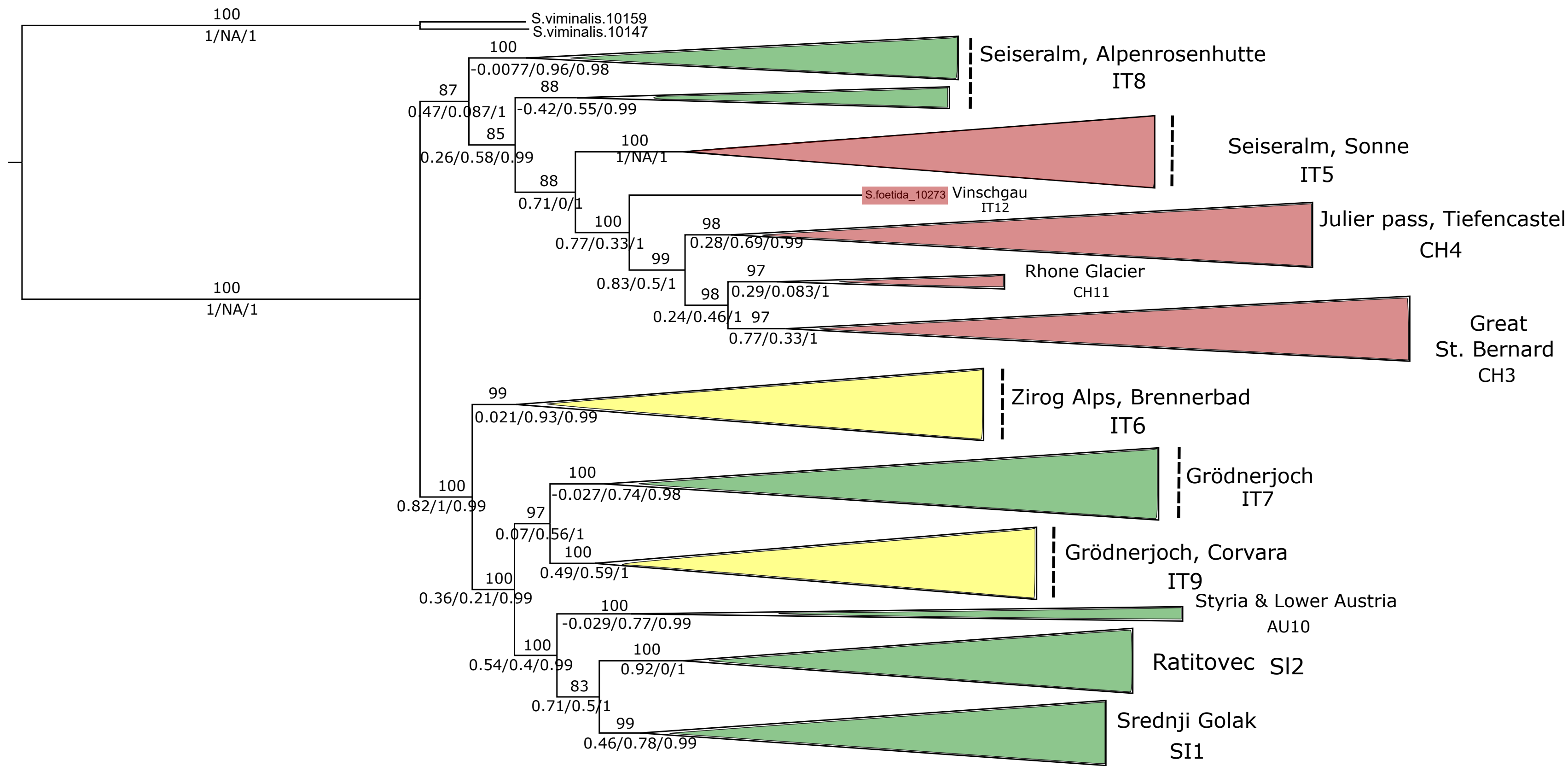

■ *S. foetida*

■ Hybrid

■ *S. waldsteiniana*

----- Contact zone

Supplement: Supplementary file 1 — Appendix S1. [file ECE3-13-e9700-s003.pdf]

K = 2

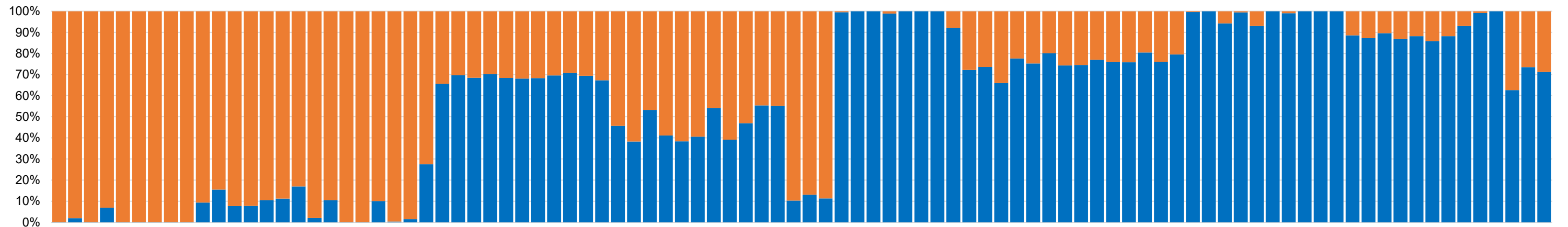

Phenotype

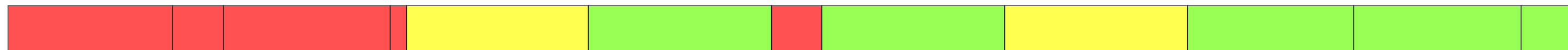

-----

Contact zone

■ *S. foetida*    ■ Hybrid    ■ *S. waldsteiniana*

Supplement: Supplementary file 2 — Appendix S2. [file ECE3-13-e9700-s002.pdf]

K = 2

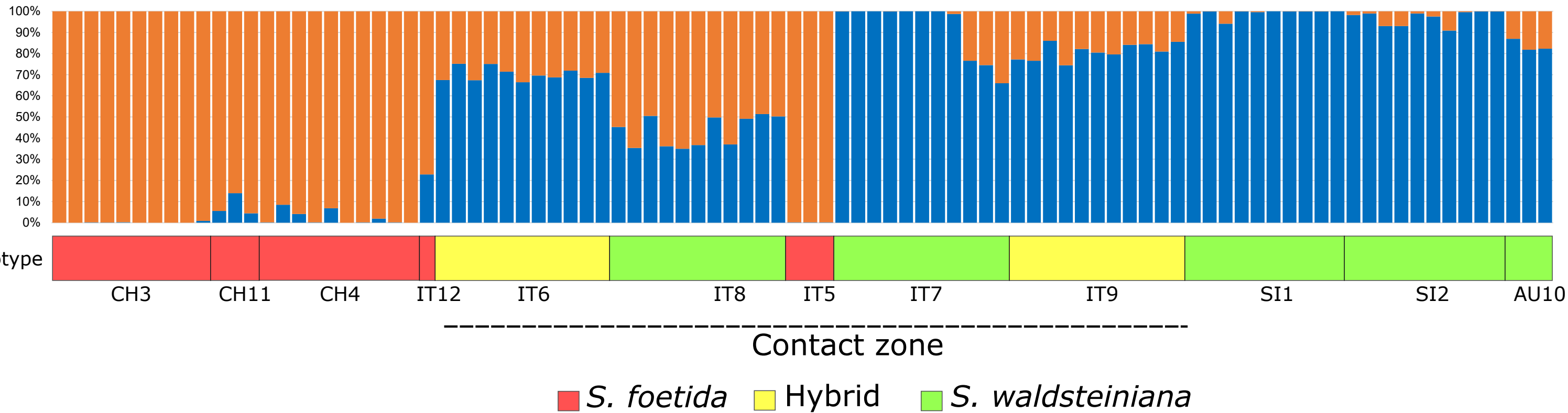

Supplement: Supplementary file 3 — Appendix S3. [file ECE3-13-e9700-s001.pdf]

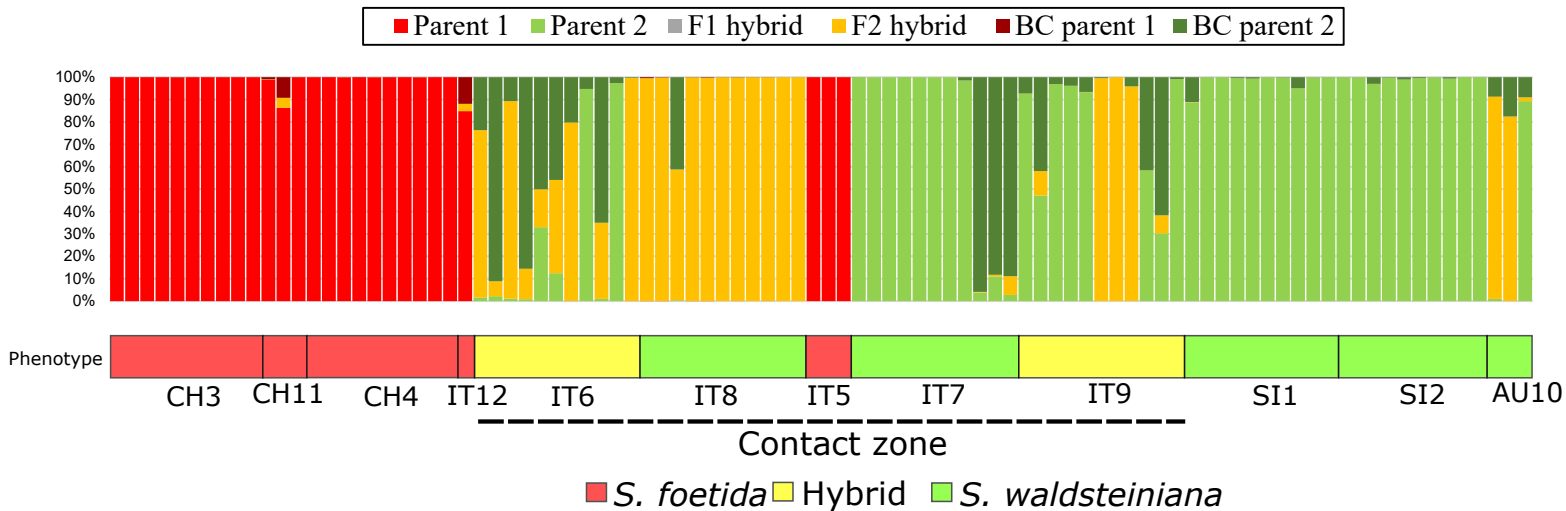

Supplement: Supplementary file 4 — Appendix S4. [file ECE3-13-e9700-s004.pdf]
